# Dronedarone provides effective early rhythm control: post-hoc analysis of the ATHENA trial using EAST-AFNET 4 criteria

**DOI:** 10.1093/europace/euaf080

**Published:** 2025-04-29

**Authors:** Paulus Kirchhof, A John Camm, Harry J G M Crijns, Jonathan P Piccini, Christian Torp-Pedersen, David S McKindley, Mattias Wieloch, Stefan H Hohnloser

**Affiliations:** Department of Cardiology, University Heart and Vascular Center Hamburg, University Medical Center Hamburg–Eppendorf, Hamburg, Germany; German Center for Cardiovascular Research, Partner Site Hamburg/Kiel/Lübeck, Germany; Institute of Cardiovascular Sciences, University of Birmingham, Birmingham, UK; Institute of Molecular and Clinical Sciences, St. George’s University of London, London, UK; Maastricht University Medical Centre and Cardiovascular Research Institute, Maastricht, The Netherlands; Duke University Medical Center and the Duke Clinical Research Institute, Durham, NC, USA; Department of Public Health, University of Copenhagen, Copenhagen, Denmark; Department of Cardiology, Nordjællands Hospital, Hillerød, Denmark; Sanofi, Bridgewater, NJ, USA; Sanofi, Paris, France; Lund University, Lund, Sweden; Department of Cardiology, J.W. Goethe University, Theodor-W.-Adorno-Platz 1, 60629 Frankfurt am Main, Germany

**Keywords:** Atrial fibrillation, Atrial flutter, Early rhythm control, Dronedarone

## Abstract

**Aims:**

This post-hoc analysis of the ATHENA trial assessed whether dronedarone (400 mg twice daily) improved cardiovascular outcomes compared with placebo in patients with early atrial fibrillation/atrial flutter (AF) and cardiovascular comorbidities, based on EAST-AFNET 4 inclusion criteria and outcomes.

**Methods and results:**

The co-primary outcomes were (i) a composite of cardiovascular death, stroke, or hospitalisation due to worsening of heart failure (HF) or acute coronary syndrome (ACS) and (ii) nights spent in hospital per year. Sinus rhythm (SR) at 12 months was a secondary outcome. The primary safety outcome was a composite of death, stroke, or pre-specified serious adverse events of special interest (AESIs) related to rhythm control therapy. 1810 patients with early AF were identified. Patients receiving dronedarone had fewer deaths from cardiovascular causes, strokes, or hospitalisations due to worsening of HF or ACS compared with patients receiving placebo [dronedarone (*n* = 924), 87 patients with ≥1 event; placebo (*n* = 886), 117 patients with ≥1 event; hazard ratio 0.71; 95% confidence interval 0.54–0.94; *P* = 0.014]. Number of nights spent in hospital did not differ between treatment groups. More patients receiving dronedarone (69.2%) were in SR at 12 months compared with placebo (60.8%). Primary safety events comprising death, stroke, or pre-specified serious AESIs related to rhythm control therapy were not different (dronedarone vs. placebo: 60 vs. 71 patients with ≥1 event).

**Conclusion:**

These data support the use of dronedarone for early rhythm control therapy in selected patients with early AF.

**Trial registration:**

ATHENA: ClinicalTrials.gov identifier NCT00174785. EAST-AFNET 4: ClinicalTrials.gov identifier NCT01288352.

## Graphical Abstract

**Graphical Abstract:**
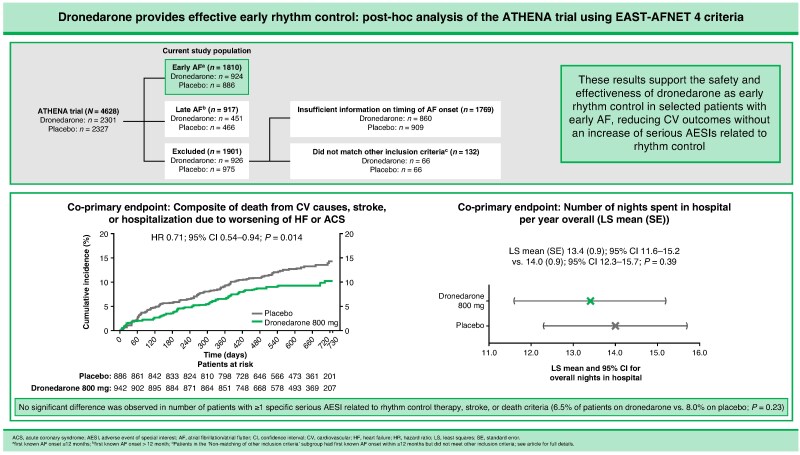

**What’s new?**

- This post-hoc analysis of the ATHENA trial assessed whether dronedarone improved cardiovascular outcomes compared with placebo in patients with recently diagnosed (also known as ‘early’) atrial fibrillation/atrial flutter and cardiovascular comorbidities, applying EAST-AFNET 4 inclusion criteria and outcomes.
- Dronedarone was associated with fewer cardiovascular deaths, strokes, and hospitalisations due to worsening of heart failure or acute coronary syndrome (EAST-AFNET 4 primary outcome) compared with placebo in eligible ATHENA patients with early atrial fibrillation/atrial flutter and cardiovascular comorbidities.
- The safety profile of dronedarone was comparable to that of placebo, and nights spent in hospital were not different between treatment groups.
- These data support the use of dronedarone as early rhythm control therapy in selected patients with atrial fibrillation/atrial flutter.

## Introduction

Despite improvements in the evaluation and management of atrial fibrillation over time, this condition remains associated with adverse clinical outcomes, including stroke, heart failure (HF), acute coronary syndrome (ACS), and death due to cardiovascular causes.^1,2^ Important aspects of atrial fibrillation therapy include anticoagulation, rate control therapy, rhythm control therapy, and identification/management of comorbid cardiovascular diseases and risk factors.^3–6^ Although antiarrhythmic drugs (AADs) are successful in maintaining sinus rhythm (SR), several AADs have been associated with increased mortality and adverse effects, including long-term toxicity.^5^

Dronedarone is a multichannel blocking benzofuran that possesses class I–IV antiarrhythmic effects.^7^ The 2020 European Society of Cardiology (ESC)/European Association for Cardio-Thoracic Surgery (EACTS) guideline for the diagnosis and management of atrial fibrillation recommends dronedarone for long-term rhythm control in atrial fibrillation patients with normal/mildly impaired (but stable) left ventricular (LV) function or HF with preserved ejection fraction (HFpEF), ischaemic heart disease, or valvular heart disease (VHD).^4^ Similarly, the 2023 American College of Cardiology (ACC)/American Heart Association (AHA)/American College of Clinical Pharmacy (ACCP)/Heart Rhythm Society (HRS) guideline for the diagnosis and management of atrial fibrillation notes the use of dronedarone for long-term SR maintenance is reasonable in patients with atrial fibrillation without recent decompensated HF or severe LV dysfunction.^8^ In the randomized, placebo-controlled ATHENA trial (NCT00174785), dronedarone reduced the incidence of unplanned hospitalisation due to cardiovascular events or death in patients with paroxysmal or persistent atrial fibrillation/atrial flutter (henceforth referred to collectively as AF) and additional risk factors for death.^9^ The Early Treatment of Atrial Fibrillation for Stroke Prevention Trial (EAST-AFNET 4; NCT01288352) later demonstrated that initiating comprehensive early rhythm control (ERC) therapy with AADs or ablation in patients with early (i.e. diagnosed within ≤12 months) AF and cardiovascular risk was associated with a lower risk of cardiovascular outcomes than guideline-based usual care over a follow-up period of >5 years.^10^

The aim of this post-hoc analysis was to evaluate the effectiveness and safety of dronedarone for ERC in AF. To achieve this, outcomes were compared in all patients randomized in the ATHENA trial who fulfilled the enrolment criteria of the EAST-AFNET 4 trial.

## Methods

### Study design

The methods for the ATHENA and EAST-AFNET 4 trials have been described previously.^9,10^ ATHENA was a randomized, placebo-controlled, international, multicenter, double-blind, parallel-group trial conducted from 2005 to 2008 that assessed the efficacy of dronedarone for prevention of unplanned cardiovascular hospitalisation or death from any cause in patients with paroxysmal or persistent AF and additional cardiovascular risk factors.^9^ EAST-AFNET 4 was a randomized, European, multicenter, parallel-group, open-labelled treatment assignment, blinded-outcome-assessment trial (2011–2016) that assessed the efficacy of comprehensive ERC vs. guideline-based usual care in patients with early AF and additional cardiovascular risk factors.^10,11^ Detailed patient eligibility criteria for the ATHENA and EAST-AFNET 4 trials are shown in Supplementary material online, *Table S1*. Patients randomized in ATHENA and fulfilling the inclusion criteria of EAST-AFNET 4 (namely first diagnosis of AF within ≤12 months and presence of ≥2 stroke risk factors) were identified and described as having early AF; patients randomized in ATHENA who had first known AF onset >12 months were described as having late AF (presence/absence of other inclusion criteria was not assessed). The effectiveness and safety of dronedarone were compared with that of placebo.

### Study outcomes

Outcomes were modelled on the primary outcomes of the EAST-AFNET 4 trial. The first co-primary outcome was a composite of death from cardiovascular causes, stroke, or hospitalisation due to worsening of HF or ACS, while the second co-primary outcome was the number of nights spent in hospital per year (with nights in hospital due to cardiovascular causes as a secondary outcome). Other secondary outcomes were the individual components of the first co-primary outcome and SR at 12 months (SR at 24 months is not presented since relatively few ATHENA trial patients remained in the study at that time point). The primary safety outcome was a composite of death from any cause, stroke, or pre-specified serious adverse events of special interest (AESIs) related to rhythm control therapy. Specific details of study definitions and pre-specified AESIs for the ATHENA and EAST-AFNET 4 trials are shown in Supplementary material online, *Table S2*.

### Statistical analysis

Log-rank testing and Cox regression were used to compare the event and hazard rates between the treatment groups. The survival data were plotted as Aalen-Johansen curves (as done for the EAST-AFNET 4 trial)^10^ to estimate the cumulative probability of being alive and reaching an endpoint.^12^ The individual components of the first co-primary outcome were assessed for statistical significance between treatment groups. An interaction test was performed by combining the ≤12 months and >12 months strata to create a Cox regression model with strata and treatment as main effects and an interaction term of strata by treatment. Nights in hospital were presented as least squares mean, alongside standard error (SE). A landmark analysis was performed to show the cumulative incidence of the primary composite outcome in patients in SR at 12 months in both treatment groups, plotted as Kaplan–Meier curves. Safety data were presented using descriptive statistics, as presented in EAST-AFNET 4.

## Results

### Demographic and baseline characteristics

**Figure 1:**
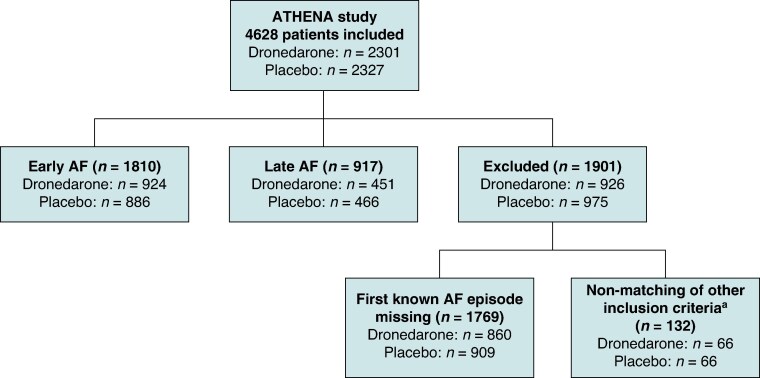
Patient disposition. Early AF defined as first known AF onset within ≤12 months. Late AF defined as first known AF onset >12 months. ^a^Patients in the ‘Non-matching of other inclusion criteria’ subgroup had first known AF onset within ≤12 months but did not meet other EAST-AFNET 4 inclusion criteria (see Supplementary material online, *Table S1* for details of these). AF, atrial fibrillation/atrial flutter.

Of 2301 patients from the dronedarone arm and 2327 patients from the placebo arm, 1810 patients were categorized as having early AF (924 and 886, respectively) and 917 patients as having late AF (451 and 466, respectively) when entering the trial. A total of 1901 patients either had insufficient information on timing of AF onset (860 in the dronedarone arm and 909 in the placebo arm) or did not match other EAST-AFNET 4 inclusion criteria such as age (66 patients) or stroke risk (66 patients). Details of other inclusion criteria are shown in Supplementary material online, *Table S1*. Patient disposition is shown in *Figure 1*.

Median time from first known AF episode to randomisation was similar in both treatment groups (50.0 days for dronedarone and 50.5 days for placebo). Demographic and baseline characteristics in patients with early AF were similar between the treatment groups. Approximately half of all patients were male, and the majority were Caucasian (>85% in both treatment groups). Over 85% were hypertensive with a mean CHA_2_DS_2_VASc score of 3.8 (*Table 1*). Approximately 26–29% of patients in each treatment group had a history of stable HF, and 76 (8.3%)/106 (12.2%) patients receiving dronedarone/placebo, respectively, had HF with reduced ejection fraction [HFrEF; defined per ACC/AHA/Heart Failure Society of America (HFSA) and ESC guidelines].^13^ The use of oral anticoagulants was similar at baseline in the dronedarone and placebo groups, with 58.1% and 58.9% of patients, respectively, using vitamin K antagonists (VKAs).

**Table 1:** Demographic and baseline characteristics, cardiovascular history, and medication history of patients with early AF (onset ≤12 months)

Characteristic	Dronedarone (*n* = 924)	Placebo (*n* = 886)
**Demographic and baseline characteristics/cardiovascular history**		
Age, mean (SD), years	73 (8.2)	73 (8.8)
Male	421 (45.6)	453 (51.1)
Race		
Caucasian	790 (85.5)	754 (85.1)
Asian	72 (7.8)	61 (6.9)
Black	13 (1.4)	21 (2.4)
Other	49 (5.3)	50 (5.6)
BMI ≥30 kg/m^2^	291 (31.5)	278 (31.4)
Hypertension	815 (88.2)	760 (85.8)
Non-insulin-dependent diabetes mellitus	180 (19.5)	182 (20.5)
Insulin-dependent diabetes mellitus	30 (3.2)	35 (4.0)
eGFR MDRD, mean (SD), mL/min	66.6 (18.4)	66.3 (19.2)
Time since first AF, median (Q1, Q3), days	50.0 (14.5, 114.5)	50.5 (14.0, 122.0)
LVEF, mean (SD), %	58.2 (11.0)	57.1 (12.0)
LVEF, %		
<35%	29 (3.2)	44 (5.1)
≤40% (i.e. HFrEF)^a^	76 (8.3)	106 (12.2)
<45%	97 (10.6)	129 (14.9)
<50%	148 (16.2)	173 (20.0)
Left HF, NYHA class		
Class I	81 (8.8)	60 (6.8)
Class II	128 (13.9)	165 (18.6)
Class III	27 (2.9)	29 (3.3)
No HF	688 (74.5)	632 (71.3)
LAD >40 mm	593 (65.0)	569 (65.9)
CHA_2_DS_2_VASc score, mean (SD)	3.8 (1.4)	3.8 (1.4)
Structural heart disease	500 (54.4)	533 (60.7)
Coronary heart disease	260 (28.1)	276 (31.2)
Non-rheumatic valvular heart disease	118 (12.8)	139 (15.7)
Pacemaker	63 (6.8)	56 (6.3)
Ablation for AF	10 (1.1)	18 (2.0)
**Medication use**		
ACE inhibitors or angiotensin II receptor antagonist	638 (69.0)	596 (67.3)
Beta-blockers (except sotalol)	638 (69.0)	601 (67.8)
Calcium antagonists with HR-lowering effects	142 (15.4)	107 (12.1)
Oral anticoagulants	537 (58.1)	522 (58.9)
Diuretics (excluding spironolactone)	473 (51.2)	469 (52.9)
Low-dose aspirin (≤365 mg)	441 (47.7)	386 (43.6)
Statins (CYP3A4 metabolized)	299 (32.4)	305 (34.4)
Digitalis	121 (13.1)	127 (14.3)

Data are *n* (%) unless otherwise stated and include the total number of patients in dronedarone and placebo groups. Early AF defined as first known AF onset within ≤12 months.

ACC, American College of Cardiology; ACE, angiotensin-converting enzyme; AF, atrial fibrillation/atrial flutter; AHA, American Heart Association; BMI, body mass index; CHA_2_DS_2_VASc, congestive heart failure, high blood pressure, age >75 years, diabetes, previous stroke or clot, vascular disease, age 65–74 years, sex; CYP3A4, cytochrome P450 3A4; eGFR, estimated glomerular filtration rate; ESC, European Society of Cardiology; HF, heart failure; HFrEF, heart failure with reduced ejection fraction; HFSA, Heart Failure Society of America; HR, heart rate; LAD, left atrium diameter; LVEF, left ventricular ejection fraction; MDRD, modification of diet in renal disease; NYHA, New York Heart Association; SD, standard deviation.

^a^Defined per ACC/AHA/HFSA 2022 and ESC 2021 guidelines.^13^

### Co-primary outcomes

**Figure 2:**
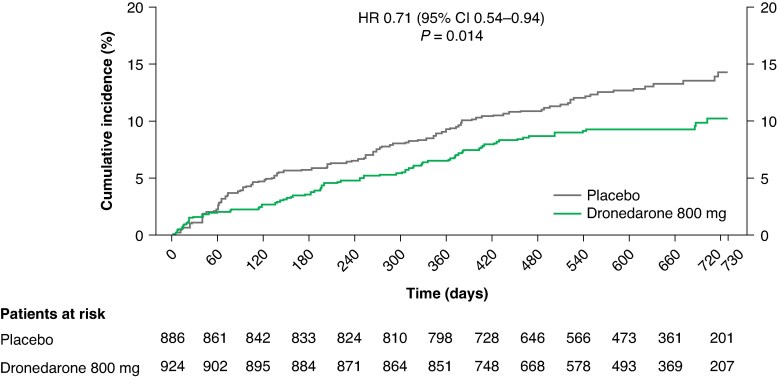
Primary composite outcome (death from cardiovascular causes, stroke, or hospitalisation due to worsening of HF or ACS) for dronedarone vs. placebo in patients with early AF. Aalen-Johansen cumulative incidence curves are shown. Early AF defined as first known AF onset within ≤12 months. ACS, acute coronary syndrome; AF, atrial fibrillation/atrial flutter; CI, confidence interval; HF, heart failure; HR, hazard ratio. Figure reproduced with permission from European Society of Cardiology.

Patients randomized to dronedarone had fewer cardiovascular events than those randomized to placebo [dronedarone: 87 patients with ≥1 event, 8.64 events/100 patient-years; placebo: 117 patients with ≥1 event, 11.62 events/100 patient-years; unadjusted hazard ratio (HR) 0.71; 95% confidence interval (CI) 0.54–0.94; *P* = 0.014; *Figure 2*]. For the second co-primary outcome, patients in the dronedarone and placebo groups spent a similar number of nights in hospital overall [least squares mean (SE): 13.4 (0.9); 95% CI 11.6–15.2 vs. 14.0 (0.9); 95% CI 12.3–15.7; *P* = 0.39; Supplementary material online, *Figure S1*].

### Safety

**Figure 3:**
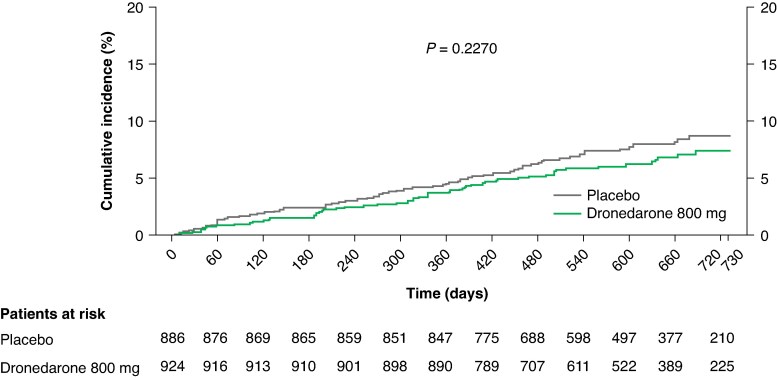
Primary safety outcome (any death, stroke, or pre-specified serious AESIs related to rhythm control therapy) for dronedarone vs. placebo in patients with early AF. Kaplan–Meier cumulative incidence curves are shown. Early AF defined as first known AF onset within ≤12 months. AESI, adverse event of special interest; AF, atrial fibrillation/atrial flutter.

For the primary safety outcome, the number of patients with ≥1 pre-specified serious AESI related to rhythm control therapy, stroke, or all-cause death was 60 (6.5%) with dronedarone and 71 (8.0%) with placebo (*P* = 0.23; *Table 2*; *Figure 3*).

**Table 2:** Outcomes for the primary safety endpoint in patients with early AF

	Dronedarone (*n* = 924)	Placebo (*n* = 886)
**Total number of patients with first composite among any death, stroke, or pre-specified serious AESIs related to rhythm control therapy^a^**	60 (6.5)	71 (8.0)
Death	53 (5.7)	59 (6.7)
Stroke	10 (1.1)	11 (1.2)
Pre-specified serious AESIs related to rhythm control therapy	2 (0.2)	4 (0.5)
**Total number of events among any death, stroke, or pre-specified serious AESIs related to rhythm control therapy/number of patients with events (ratio)**	85/60 (1.4)	99/71 (1.4)

Data are *n* (%) and include the total number of patients in dronedarone and placebo groups. Early AF defined as first known AF onset within ≤12 months. Patients were included in one category only, in the following order of priority: death, stroke, pre-specified AESIs related to rhythm control therapy.

AESI, adverse event of special interest; AF, atrial fibrillation/atrial flutter.

^a^Pre-specified AESIs related to rhythm control therapy are listed in Supplementary material online, *Table S2*, aligned with the EAST-AFNET 4 primary safety composite endpoint.

### Secondary outcomes, comparison to late AF

Statistical analyses of the individual components of the primary composite outcome are described in *Table 3*. Numerically fewer patients assigned to dronedarone experienced any individual component of the primary composite outcome compared with patients assigned to placebo. At 12 months of follow-up, 69.2% (*n*/*N* = 639/924) of patients assigned to dronedarone were in SR, a higher proportion than the 60.8% (*n/N* = 539/886) of patients assigned to placebo. For nights in hospital due to cardiovascular causes, patients on dronedarone spent 10.3 (1.0; 95% CI 8.2–12.3) nights in hospital compared with 12.0 (0.9; 95% CI 10.1–13.8) nights for patients on placebo [least squares mean (SE)]. Patients in SR at 12 months who were treated with dronedarone were numerically less likely to experience the primary composite outcome compared with patients treated with placebo (see Supplementary material online, *Figure S2A*). Conversely, patients who were not in SR at 12 months (i.e. still experiencing AF) who were treated with placebo were numerically less likely to experience the primary composite outcome compared with patients treated with dronedarone (see Supplementary material online, *Figure S2B*).

**Table 3:** Outcomes of the individual components of the primary composite endpoint for patients with early AF

	Number of patients with ≥1 event (%)		HR (95% CI)	*P* value
	Dronedarone (*n* = 924)	Placebo (*n* = 886)		
Cardiovascular death	29 (1.6)	41 (2.3)	0.68 (0.42–1.09)	0.110
Stroke	10 (0.6)	11 (0.6)	0.88 (0.37–2.07)	0.768
HF hospitalisation	48 (2.7)	55 (3.0)	0.84 (0.57–1.24)	0.377
ACS hospitalisation	16 (0.9)	35 (1.9)	0.44 (0.24–0.79)	0.005

Early AF defined as first known AF onset within ≤12 months.

ACS, acute coronary syndrome; AF, atrial fibrillation/atrial flutter; CI, confidence interval; HF, heart failure; HR, hazard ratio.

In patients with late AF (for whom demographic and baseline characteristics are shown in Supplementary material online, *Table S3*), similar, albeit non-significant, results were observed for the primary composite outcome (unadjusted HR 0.79; 95% CI 0.54–1.14; *P* = 0.206; Supplementary material online, *Figure S3*) and for secondary outcomes (see Supplementary material online, *Table S4*). There was no interaction between treatment group and early or late AF presentation (*P* = 0.641). Percentages of safety outcomes were at the same levels in patients with late AF receiving either dronedarone or placebo (see Supplementary material online, *Table S5*).

## Discussion

Results from this post-hoc analysis of the ATHENA trial show that dronedarone improved cardiovascular outcomes compared with placebo in patients with recently diagnosed AF and cardiovascular risk factors. These results replicate the main findings from the EAST-AFNET 4 trial^10^ and support the use of dronedarone as ERC therapy.

Subanalyses of the EAST-AFNET 4 trial showed a consistent effect extended to patients with HF with reduced, mid-range, or preserved ejection fraction (*n* = 798) and also to asymptomatic patients (*n* = 801).^11,14^ Dronedarone was not evaluated independently in EAST-AFNET 4, but was one of the most common AADs used to initiate ERC in that trial; 16.7% of patients randomized to ERC were initially treated with dronedarone.^10^ A mediator analysis of the EAST-AFNET 4 trial demonstrated that achieving SR was the key mediator of effective ERC; the type of rhythm control therapy, including AF ablation, had a lesser effect. In EAST-AFNET 4, ERC was commonly started with an AAD.^10,15^

Patients in the ATHENA post-hoc analysis were slightly older than patients in the EAST-AFNET 4 trial, more likely to be female and had higher CHA_2_DS_2_-VASc scores; the proportion of patients with BMI ≥30 kg/m^2^ was also slightly higher.^10^ Furthermore, a lower proportion of patients in the ATHENA post-hoc analysis had HF, while a higher proportion had pacemakers installed compared with the EAST-AFNET 4 trial population.^10^ Median time since first AF was longer in ATHENA post-hoc analysis (by ∼2 weeks) than in the EAST AFNET-4 trial.^10^ Approximately 90% of patients in EAST-AFNET 4 received anticoagulation at baseline [either direct oral anticoagulants (DOACs) or VKAs]^10^ compared with ∼60% of patients in this ATHENA post-hoc analysis (VKAs only); this is explained by the non-availability of DOACs when the ATHENA trial was conducted, less awareness about the benefits of anticoagulation vs. risk of bleeding, and changes in the recommendations for stroke prevention during this time frame compared with current guidelines. Although there were differences in their use, anticoagulant treatment was balanced between treatment groups in this analysis, unlike the withdrawal of oral anticoagulation in the AFFIRM trial that probably contributed to the neutral outcome of that trial,^16^ even in patients with recently diagnosed atrial fibrillation.^17^

Nights spent in hospital did not differ between treatment groups in this analysis, similar to the neutral effect of ERC on the same outcome in the EAST-AFNET 4 trial.^10^ Study drug was initiated as outpatient treatment in ATHENA,^9^ similar to the outpatient initiation of AADs in most centres in the EAST-AFNET 4 trial.^15^ A recent subanalysis of the EAST-AFNET 4 trial found that SR at 12 months was the most relevant mediator of ERC effectiveness and was responsible for 81% of treatment effect vs. usual care during the 4.1-year follow-up period.^18^ In the current analysis, a greater proportion of patients receiving dronedarone were in SR at 12 months compared with patients receiving placebo (69.2% vs. 60.8%), and patients on dronedarone who were in SR at 12 months were less likely than those on placebo to subsequently experience the primary composite outcome. It appears that surviving patients who were in SR at 12 months benefitted significantly from dronedarone in terms of event-free survival. This may possibly relate to better prevention of atrial fibrillation recurrence with dronedarone vs. placebo. In contrast, patients who received dronedarone and were experiencing atrial fibrillation at 12 months displayed a higher incidence of the primary composite outcome vs. placebo. Dronedarone has a well-documented rhythm-controlling effect in patients with atrial fibrillation.^19–22^ While the findings of this analysis suggest that attaining SR mediates in part the effect of ERC, precise data on recurrent atrial fibrillation are not available in ATHENA, and so these findings are to be considered hypothesis-generating in nature.

It should be noted that the majority of patients (85.3%) were Caucasian, which may limit generalizability of these results. However, the efficacy and tolerability of dronedarone in the Asian population of ATHENA (7.3% of patients) were previously assessed and found to be consistent with results observed in the non-Asian population,^23^ suggesting the results of the current analysis may be relevant to other populations. In other ATHENA subanalyses, dronedarone has been associated with reduced progression to permanent AF and increased regression to SR vs. placebo.^24^ Risk of cardiovascular hospitalisation or death due to any cause was similarly reduced with dronedarone vs. placebo independent of sex and was also reduced with dronedarone vs. placebo in patients aged <65 or ≥65 years, with a greater effect in the latter group (although incidence of treatment-emergent adverse events was also higher in the older patient population).^25^ Additionally, real-world data shows that dronedarone is as effective as other AADs (i.e. amiodarone, flecainide, propafenone, and sotalol) in patients with atrial fibrillation^26^ and has been suggested as a potentially safer/more effective post-ablation treatment option vs. sotalol based on a comparative analysis of the two agents.^27^

The present analysis does have limitations. First, this is a post-hoc analysis, and as mentioned, the ATHENA trial did not primarily evaluate rhythm control with dronedarone vs. placebo, nor ERC *per se*. Hence, a significant number of patients in the ATHENA trial did not fit the EAST-AFNET 4 inclusion criteria (most commonly due to lack of information regarding timing of AF onset) and were therefore excluded from this analysis. However, the sample size is still one of the largest seen in a placebo-controlled trial of an AAD. Furthermore, some adverse safety events of interest in EAST-AFNET 4, like hospitalisation for atrial fibrillation (pro-arrhythmia) or HF (worsening after AAD initiation), could not be validated in this analysis and have hence been excluded. Second, the nature of the control group varied between ATHENA and EAST-AFNET 4; in ATHENA, dronedarone was compared with placebo, whilst in EAST-AFNET 4, ERC was compared with usual care including symptom-directed therapy with AADs and AF ablation. Third, as previously mentioned, the proportion of patients treated with VKAs (e.g. warfarin) in ATHENA was lower than that of more recent trials such as EAST-AFNET 4, which also included anticoagulation with DOACs. Changes in the management of atrial fibrillation (and the rise in OAC use with the advent of DOACs) since the ATHENA trial was conducted may mean that dronedarone could have a different effect in contemporary patients with atrial fibrillation compared with the ATHENA population. Furthermore, this analysis cannot answer the question of whether dronedarone, or other types of rhythm control therapy, are effective when initiated late after a first diagnosis of AF. While no interaction was observed between randomized treatment and early or late AF diagnosis, this analysis was underpowered as only 917 patients had late AF. Finally, the definitions of the outcomes evaluated in this analysis differ from those specified in the ATHENA trial (details available in Supplementary material online, *Table S2*), and thus, the interpretation of these results may vary across different patient subgroups.

Overall, the results from this post-hoc analysis of the ATHENA trial using EAST-AFNET 4 inclusion criteria and outcomes support the effectiveness and safety of dronedarone compared with placebo for ERC in patients with early AF, reducing cardiovascular outcomes without an increase in serious AESIs related to rhythm control therapy according to EAST-AFNET 4 criteria. Further data are needed to evaluate the outcome-reducing effect of rhythm control therapy in patients with longer durations of AF.

## Supplementary Material

euaf080_Supplementary_Data

## Data Availability

Qualified researchers may request access to patient-level data and related study documents, including the clinical study report, study protocol with any amendments, blank case report form, statistical analysis plan, and dataset specifications. Patient-level data will be anonymized and study documents will be redacted to protect the privacy of our trial participants. Further details on Sanofi’s data-sharing criteria, eligible studies, and process for requesting access can be found at https://www.vivli.org/.

## References

[1] Desai NR, Giugliano RP. Can we predict outcomes in atrial fibrillation? Clin Cardiol 2012;35:10–4. 22246946 10.1002/clc.20989 PMC6652364

[2] Marijon E, Le Heuzey JY, Connolly S, Yang S, Pogue J, Brueckmann M et al Causes of death and influencing factors in patients with atrial fibrillation: a competing-risk analysis from the randomized evaluation of long-term anticoagulant therapy study. Circulation 2013;128:2192–201. 24016454 10.1161/CIRCULATIONAHA.112.000491

[3] National Institute for Health and Care Excellence . *Atrial fibrillation: diagnosis and management 2021*. Available from: https://www.nice.org.uk/guidance/ng196/chapter/Recommendations (30 January 2025, date last accessed). 34165935

[4] Hindricks G, Potpara T, Dagres N, Arbelo E, Bax JJ, Blomström-Lundqvist C et al 2020 ESC guidelines for the diagnosis and management of atrial fibrillation developed in collaboration with the European Association for Cardio-Thoracic Surgery (EACTS): the task force for the diagnosis and management of atrial fibrillation of the European Society of Cardiology (ESC) developed with the special contribution of the European Heart Rhythm Association (EHRA) of the ESC. Eur Heart J 2021;42:373–498. 32860505 10.1093/eurheartj/ehaa612

[5] Valembois L, Audureau E, Takeda A, Jarzebowski W, Belmin J, Lafuente-Lafuente C. Antiarrhythmics for maintaining sinus rhythm after cardioversion of atrial fibrillation. Cochrane Database Syst Rev 2019;9:CD005049. 31483500 10.1002/14651858.CD005049.pub5 PMC6738133

[6] Schnabel RB, Marinelli EA, Arbelo E, Boriani G, Boveda S, Buckley CM et al Early diagnosis and better rhythm management to improve outcomes in patients with atrial fibrillation: the 8th AFNET/EHRA consensus conference. Europace 2023;25:6–27. 35894842 10.1093/europace/euac062 PMC9907557

[7] Patel C, Yan GX, Kowey PR. Dronedarone. Circulation 2009;120:636–44. 19687370 10.1161/CIRCULATIONAHA.109.858027

[8] Joglar JA, Chung MK, Armbruster AL, Benjamin EJ, Chyou JY, Cronin EM et al 2023 ACC/AHA/ACCP/HRS guideline for the diagnosis and management of atrial fibrillation: a report of the American College of Cardiology/American Heart Association Joint Committee on Clinical Practice Guidelines. Circulation 2024;149:e1–156. 38033089 10.1161/CIR.0000000000001193 PMC11095842

[9] Hohnloser SH, Crijns HJ, van Eickels M, Gaudin C, Page RL, Torp-Pedersen C et al Effect of dronedarone on cardiovascular events in atrial fibrillation. N Engl J Med 2009;360:668–78. 19213680 10.1056/NEJMoa0803778

[10] Kirchhof P, Camm AJ, Goette A, Brandes A, Eckardt L, Elvan A et al Early rhythm-control therapy in patients with atrial fibrillation. N Engl J Med 2020;383:1305–16. 32865375 10.1056/NEJMoa2019422

[11] Rillig A, Magnussen C, Ozga AK, Suling A, Brandes A, Breithardt G et al Early rhythm control therapy in patients with atrial fibrillation and heart failure. Circulation 2021;144:845–58. 34328366 10.1161/CIRCULATIONAHA.121.056323 PMC8456351

[12] Open Risk Manual . Available from: https://www.openriskmanual.org/wiki/Aalen-Johansen_Estimator (30 January 2025, date last accessed).

[13] Behnoush AH, Khalaji A, Naderi N, Ashraf H, von Haehling S. ACC/AHA/HFSA 2022 and ESC 2021 guidelines on heart failure comparison. ESC Heart Fail 2023;10:1531–44. 36460629 10.1002/ehf2.14255 PMC10192289

[14] Willems S, Borof K, Brandes A, Breithardt G, Camm AJ, Crijns HJGM et al Systematic, early rhythm control strategy for atrial fibrillation in patients with or without symptoms: the EAST-AFNET 4 trial. Eur Heart J 2022;43:1219–30. 34447995 10.1093/eurheartj/ehab593 PMC8934687

[15] Metzner A, Suling A, Brandes A, Breithardt G, Camm AJ, Crijns HJGM et al Anticoagulation, therapy of concomitant conditions, and early rhythm control therapy: a detailed analysis of treatment patterns in the EAST-AFNET 4 trial. Europace 2022;24:552–64. 34473249 10.1093/europace/euab200 PMC8982435

[16] Corley SD, Epstein AE, DiMarco JP, Domanski MJ, Geller N, Greene HL et al Relationships between sinus rhythm, treatment, and survival in the Atrial Fibrillation Follow-Up Investigation of Rhythm Management (AFFIRM) study. Circulation 2004;109:1509–13. 15007003 10.1161/01.CIR.0000121736.16643.11

[17] Yang E, Tang O, Metkus T, Berger RD, Spragg DD, Calkins HG et al The role of timing in treatment of atrial fibrillation: an AFFIRM substudy. Heart Rhythm 2021;18:674–81. 33383228 10.1016/j.hrthm.2020.12.025

[18] Eckardt L, Sehner S, Suling A, Borof K, Breithardt G, Crijns H et al Attaining sinus rhythm mediates improved outcome with early rhythm control therapy of atrial fibrillation: the EAST-AFNET 4 trial. Eur Heart J 2022;43:4127–44. 36036648 10.1093/eurheartj/ehac471 PMC9584752

[19] Goette A, Benninger G, Pittrow D, Paar WD, von Stritzky B, Bosch RF. One-year safety and quality of life outcomes in patients with atrial fibrillation on dronedarone: prospective, non-interventional study in German ambulatory care. Herzschrittmacherther Elektrophysiol 2015;26:148–54. 25750090 10.1007/s00399-015-0360-z PMC4480946

[20] Singh BN, Connolly SJ, Crijns HJ, Roy D, Kowey PR, Capucci A et al Dronedarone for maintenance of sinus rhythm in atrial fibrillation or flutter. N Engl J Med 2007;357:987–99. 17804843 10.1056/NEJMoa054686

[21] Iram F, Ali S, Ahmad A, Khan S, Husain A. A review on dronedarone: pharmacological, pharmacodynamic and pharmacokinetic profile. J Acute Dis 2016;5:102–8.

[22] Page RL, Connolly SJ, Crijns HJ, van Eickels M, Gaudin C, Torp-Pedersen C et al Rhythm- and rate-controlling effects of dronedarone in patients with atrial fibrillation (from the ATHENA trial). Am J Cardiol 2011;107:1019–22. 21296333 10.1016/j.amjcard.2010.11.028

[23] Ma C, Lin JL, Bai R, Sun Y, Nam GB, Stewart J et al Effect of dronedarone in the treatment of atrial fibrillation in the Asian population: post hoc analysis of the ATHENA trial. Clin Ther 2022;44:1203–13. 35927094 10.1016/j.clinthera.2022.07.005

[24] Blomström-Lundqvist C, Naccarelli GV, McKindley DS, Bigot G, Wieloch M, Hohnloser SH. Effect of dronedarone vs. placebo on atrial fibrillation progression: a post hoc analysis from ATHENA trial. Europace 2023;25:845–54. 36758013 10.1093/europace/euad023 PMC10062319

[25] Curtis AB, Zeitler EP, Malik A, Bogard A, Bhattacharyya N, Stewart J et al Efficacy and safety of dronedarone across age and sex subgroups: a post hoc analysis of the ATHENA study among patients with non-permanent atrial fibrillation/flutter. Europace 2022;24:1754–62. 34374766 10.1093/europace/euab208 PMC9681127

[26] Khachatryan A, Merino JL, de Abajo FJ, Botto GL, Kirchhof P, Breithardt G et al International cohort study on the effectiveness of dronedarone and other antiarrhythmic drugs for atrial fibrillation in real-world practice (EFFECT-AF). Europace 2022;24:899–909. 34792111 10.1093/europace/euab262 PMC9282916

[27] Wharton JM, Piccini JP, Koren A, Huse S, Ronk CJ. Comparative safety and effectiveness of sotalol versus dronedarone after catheter ablation for atrial fibrillation. J Am Heart Assoc 2022;11:e020506. 35060388 10.1161/JAHA.120.020506 PMC9238499

