# Supplementary material for: Dronedarone provides effective early rhythm control: post-hoc analysis of the ATHENA trial using EAST-AFNET 4 criteria
Source: Europace. 2025 Apr 29;27(4):euaf080. doi: 10.1093/europace/euaf080 (PMC12037275; doi:10.1093/europace/euaf080)
Supplement: euaf080_Supplementary_Data [file euaf080_supplementary_data.docx]

**Supplementary materials**

| **Table/Figure** | **Page** |
| --- | --- |
| Table S1 Inclusion criteria for the EAST-AFNET 4 study and ATHENA post-hoc analysis | 2 |
| Table S2 List of study definitions (including pre-specified serious AESIs related to rhythm control therapy) in EAST-AFNET 4 and ATHENA studies | 4 |
| Table S3 Demographic and baseline characteristics, cardiovascular history, and medication history of patients with late AF (onset >12 months) | 7 |
| Table S4 Efficacy results for patients with late AF | 11 |
| Table S5 Outcomes for the primary safety endpoint in patients with late AF | 12 |
| Figure S1 Overall number of nights spent in hospital per year (least squares mean and 95% CI) for dronedarone vs. placebo in patients with early AF | 13 |
| Figure S2 Landmark analysis in patients in (A) SR and (B) AF at 12 months showing cumulative incidence of primary composite outcome from month 12 for dronedarone vs. placebo in patients with early AF | 14 |
| Figure S3 Primary composite outcome (death from cardiovascular causes, stroke, or hospitalization due to worsening of HF or ACS) for dronedarone vs. placebo in patients with late AF | 16 |
| References | 17 |

**Table S1** Inclusion criteria for the EAST-AFNET 4 trial and ATHENA post-hoc analysis^1–3^

| **EAST-AFNET 4 trial inclusion criteria** | **ATHENA post-hoc analysis inclusion criteria** |
| --- | --- |
| - Recent-onset (aka early) AF (≤12 months before enrollment) - ≥1 ECG within recent 12 months that documents AF, whereas the AF episode must last longer than 30 s - One of the following: - Age >75 years - Previous transient ischemic attack or stroke   OR   - Met two of the following criteria: - Age >65 years - Female sex - Arterial hypertension (chronic treatment for hypertension, estimated need for continuous antihypertensive therapy, or resting blood pressure 145/90 mm Hg) - Diabetes mellitus (treated by drugs or insulin) - Severe coronary artery disease (previous MI, PCI, or CABG) - Peripheral artery disease - HF stable NYHA II-IIIa or LVEF <50% - Chronic kidney disease (MDRD stage 3 or 4  [GFR 15–59 mL/min/1.73 m^2^]) - Left ventricular hypertrophy (diastolic septal wall width, >15 mm) on ECG | - Early AF (≤12 months before enrollment) - Age ≥75 years - Previous transient ischemic attack or stroke   OR   - Met two of the following criteria: - Aged ≥65 years, female sex, HF, hypertension,^a^ diabetes mellitus - Severe coronary artery disease (previous MI, PCI, or CABG) - Embolism/thrombosis - HF stable NYHA II-III or LVEF <50%^b^ - Chronic kidney disease (MDRD stage 3 or 4  [GFR 15–59 mL/min/1.73 m^2^]) - No information available on left ventricular hypertrophy, but this was not an exclusion criterion |

AF, atrial fibrillation/atrial flutter; CABG, coronary artery bypass graft; ECG, electrocardiogram; GFR, glomerular filtration rate; HF, heart failure; LVEF, left ventricular ejection fraction; MDRD, modification of diet in renal disease; MI, myocardial infarction; NYHA, New York Heart Association; PCI, percutaneous coronary intervention. ^a^With ongoing therapy involving ≥2 antihypertensive drugs of different classes. ^b^Patients were not allowed to be hospitalized for HF in the last 4 weeks before randomization.

**Table S2** List of definitions (including pre-specified serious AESIs related to rhythm control therapy) in EAST-AFNET 4 and ATHENA trials

| **EAST-AFNET 4 trial definition** | **ATHENA trial definition** |
| --- | --- |
| **PRIMARY COMPOSITE EFFICACY OUTCOME (sum of cardiovascular death, hospitalization due to ACS/HF, stroke)** |  |
| Cardiovascular death | Cardiovascular death |
| Hospitalization for ACS (sum of events below) |  |
| - STEMI or NSTEMI | Acute MI |
| - Unstable angina pectoris | Unstable angina pectoris |
| - Stable angina pectoris or atypical chest pain | Stable angina (reason for hospitalization) |
| Hospitalization for HF |  |
| - Worsening of HF, decompensated OR not compensated | Worsening of HF (reason for hospitalization) |
| STROKE (sum of events below) |  |
| - TIA | TIA |
| - Ischemic stroke (including transient events with matching lesion on cerebral imaging) | Ischemic stroke |
| - Hemorrhagic stroke | Hemorrhagic stroke |
| - Stroke, other cause, or unknown cause | All stroke minus TIA, ischemic stroke, hemorrhagic stroke |
| **PRIMARY COMPOSITE SAFETY OUTCOME (sum of death, stroke, serious AESIs related to rhythm control therapy)** |  |
| Death | All-cause death |
| Stroke | See definition of stroke above |
| Serious AESIs related to rhythm control therapy | Sum of all events below (a patient can have more than one event) |
| - Torsade de pointes tachycardia | Torsade de pointes tachycardia |
| - Drug-induced bradycardia | Bradycardia or sinus bradycardia (not indicated if drug-induced) |
| - AV nodal block | AV nodal block (not indicated if drug-induced) |
| - Drug toxicity of AF-related drug therapy | Composite of serious adverse events of interstitial lung disease, liver toxicity events, and thyroid toxicity (hyperthyroidism/hypothyroidism) |
| - Non-fatal cardiac arrest | Cardiac arrest (without mortality event) or ventricular tachycardia or ventricular fibrillation |
| - Other events related to rhythm control therapy |  |
| - Blood pressure related (hypotension, hypertension; except syncope) | Hypotension/hypertension as reason for cardiovascular hospitalization |
| - Implantation of a pacemaker, ICD, CRT, or any other cardiac device | Implantation as reason for cardiovascular hospitalization |
| - Syncope | Syncope as reason for cardiovascular hospitalization |
| - Other cardiovascular event | Hypovolemic shock, cardiac transplantation, cardiovascular surgery as reason for cardiovascular hospitalization |
| Hospitalization for atrial fibrillation | Not included in safety endpoint, as atrial fibrillation-related pro-arrhythmia could not be differentiated in ATHENA, and AF post-ablation is not relevant for this analysis |
| Hospitalization for worsening of HF with decompensated HF | Not included in safety endpoint, as worsening of HF due to rhythm control therapy could not be verified |

ACS, acute coronary syndrome; AESI, adverse event of special interest; AF, atrial fibrillation/atrial flutter; AV, atrioventricular; CRT, cardiac resynchronization therapy; HF, heart failure; ICD, implantable cardioverter defibrillator; MI, myocardial infarction; NSTEMI, non–ST-segment elevation myocardial infarction; STEMI, ST-segment elevation myocardial infarction; TIA, transient ischemic attack.

**Table S3** Demographic and baseline characteristics, cardiovascular history, and medication history of patients with late AF (onset >12 months)

| **Characteristic** | **Dronedarone**  **(*n* = 451)** | **Placebo**  **(*n* = 466)** |  |
| --- | --- | --- | --- |
| **Demographic and baseline characteristics/cardiovascular history** |  |  |  |
| Age, mean (SD), years | 71.5 (9.0) | 71.7 (8.4) |  |
| Male | 244 (54.1) | 274 (58.8) |  |
| Race  Caucasian  Asian  Black  Other | 403 (89.4)  36 (8.0)  3 (0.7)  9 (2.0) | 403 (86.5)  50 (10.7)  3 (0.6)  10 (2.1) | |
| BMI ≥30 kg/m^2^ | 140 (31.0) | 125 (26.8) |  |
| Hypertension | 375 (83.1) | 395 (84.8) |  |
| Non–insulin-dependent diabetes mellitus | 91 (20.2) | 70 (15.0) |  |
| Insulin-dependent diabetes mellitus | 8 (1.8) | 16 (3.4) |  |
| eGFR MDRD, mean (SD), mL/min | 68.8 (28.1) | 67.8 (19.1) |  |
| Time since first AF, median (Q1, Q3), days | 1401 (806.0, 2463.0) | 1336 (793.0, 2383.0) |  |
| LVEF, mean (SD), % | 56.3 (11.0) | 56.6 (12.2) |  |
| LVEF, %  <35%  ≤40% (i.e.HFrEF)*  <45%  <50% | 21 (4.8)  45 (10.3)  58 (13.2)  91 (20.8) | 24 (5.3)  52 (11.4)  65 (14.3)  96 (21.1) |  |
| Left HF, NYHA class  Class I  Class II  Class III | 33 (7.3)  71 (15.7)  14 (3.1) | 37(7.9)  74 (15.9)  19 (4.1) |  |
| No HF | 333 (73.8) | 336 (72.1) |  |
| LAD >40 mm | 308 (70.3) | 339 (73.9) |  |
| CHA_2_DS_2_VASc score, mean (SD) | 3.7 (1.6) | 3.6 (1.6) |  |
| Structural heart disease | 271 (60.9) | 289 (62.6) |  |
| Coronary heart disease | 141 (31.3) | 168 (36.1) |  |
| Non-rheumatic valvular heart disease | 74 (16.4) | 69 (14.8) |  |
| Pacemaker | 48 (10.6) | 65 (13.9) |  |
| Ablation for atrial fibrillation | 32 (7.1) | 24 (5.2) |  |
| **Medication use** |  |  |  |
| ACE inhibitors or angiotensin II receptor antagonist | 311 (69.0) | 315 (67.6) |  |
| Beta-blockers (except sotalol) | 296 (65.6) | 328 (70.4) |  |
| Calcium antagonists with HR-lowering effects | 83 (18.4) | 72 (15.5) |  |
| Oral anticoagulants | 311 (69.0) | 311 (66.7) |  |
| Diuretics (excluding spironolactone) | 228 (50.6) | 238 (51.1) |  |
| Low-dose aspirin (≤365 mg) | 175 (38.8) | 194 (41.6) |  |
| Statins (CYP3A4 metabolized) | 159 (35.3) | 166 (35.6) |  |
| Digitalis | 77 (17.1) | 72 (15.5) |  |

*Defined as per ACC/AHA/HFSA 2022 and ESC 2021 guidelines.

ACC, American College of Cardiology; ACE, angiotensin-converting enzyme; AF, atrial fibrillation/atrial flutter; AHA, American Heart Association; BMI, body mass index; CHA_2_DS_2_VASc, congestive heart failure, high blood pressure, age >75 years, diabetes, previous stroke or clot, vascular disease, age 65–74 years, sex; CYP3A4, cytochrome P450 3A4; eGFR, estimated glomerular filtration rate; ESC, European Society of Cardiology; HF, heart failure; HFrEF, heart failure with reduced ejection fraction; HFSA, Heart Failure Society of America; HR, heart rate; LAD, left atrium diameter; LVEF, left ventricular ejection fraction; MDRD, modification of diet in renal disease; NYHA, New York Heart Association; SD, standard deviation. Late AF defined as first known AF onset >12 months.

**Table S4** Efficacy results for patients with late AF

|  | **Dronedarone**  ***n* = 451** | **Placebo**  ***n* = 466** |
| --- | --- | --- |
| **Primary composite outcome (death from cardiovascular causes, stroke, or hospitalization due to worsening of HF or ACS)** | | |
| Unadjusted HR (95% CI); *P* value | 0.79 (0.54–1.14); *P* = 0.21 | |
| **Number of nights spent in hospital, least squares mean (SE)** | | |
| Overall | 13.9 (1.3) | 16.3 (1.2) |
| Cardiovascular reasons | 12.2 (1.4) | 13.7 (1.2) |
| **Number (%) in SR at 12 months** | 240 (53.2) | 222 (47.6) |

ACS, acute coronary syndrome; AF, atrial fibrillation/atrial flutter; CI, confidence interval; ECG, electrocardiogram; HF, heart failure; HR, hazard ratio; SE, standard error; SR, sinus rhythm.

ECG rhythm status determined at month 12. Late AF defined as first known AF onset >12 months.
Death from cardiovascular causes, stroke, or hospitalization due to worsening of HF or ACSs.

**Table S5** Outcomes for the primary safety endpoint in patients with late AF

|  | **Dronedarone**  (*n* = 451) | **Placebo**  (*n* = 466) |
| --- | --- | --- |
| **Total number of patients with first composite among any death, stroke, or pre-specified serious AESIs related to rhythm control therapy^a^** | 35 (7.8) | 35 (7.5) |
| Death | 25 (5.5) | 24 (5.2) |
| Stroke | 13 (2.9) | 12 (2.6) |
| Pre-specified serious AESIs related to rhythm control therapy | 1 (0.2) | 0 (0.0) |
| **Total number of events among any death, stroke, or pre-specified serious AESIs related to rhythm control therapy/number of patients with events (ratio)** | 64/35 (1.8) | 55/35 (1.6) |

AESI, adverse event of special interest; AF, atrial fibrillation/atrial flutter.
Data are n (%) and include the total number of patients in dronedarone and placebo groups. Late AF defined as first known AF onset >12 months. Patients were included in one category only, in the following order of priority: death, stroke, pre-specified AESIs related to rhythm control therapy.  ^a^Pre-specified AESIs related to rhythm control therapy are listed in Supplementary material online, Table S2, aligned with the EAST-AFNET 4 primary composite safety outcome.


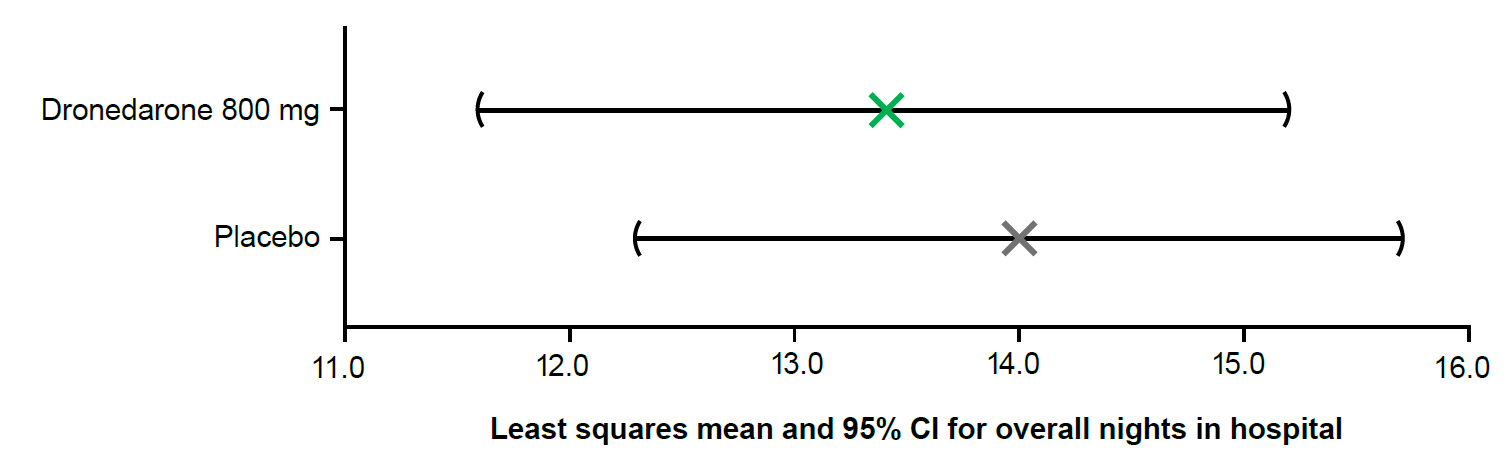


**Figure S1** Overall number of nights spent in hospital per year (least squares mean and 95% CI) for dronedarone vs. placebo in patients with early AF. Early AF defined as first known AF onset within ≤12 months. AF, atrial fibrillation/atrial flutter; CI, confidence interval.

**A**

**
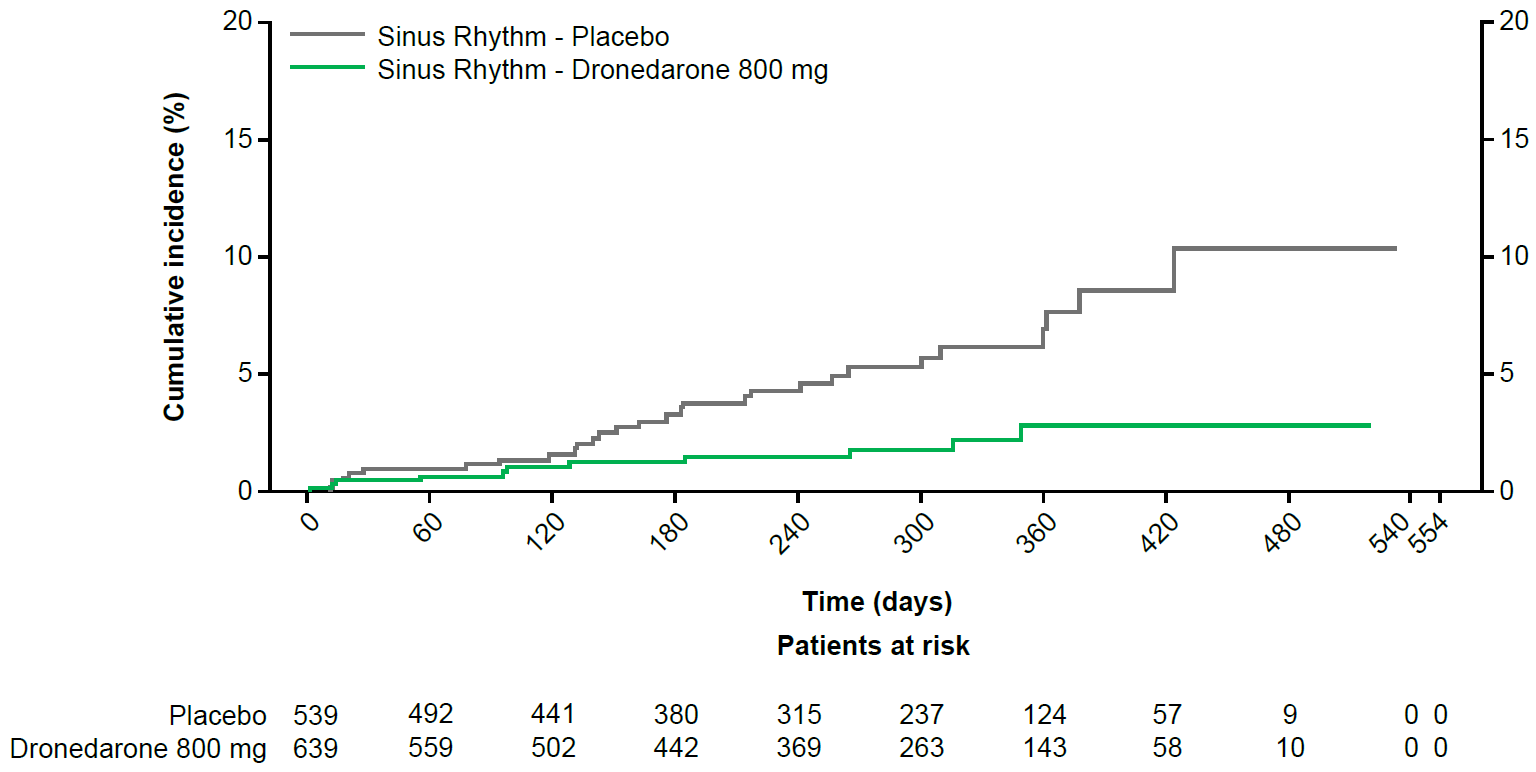
**

**B**


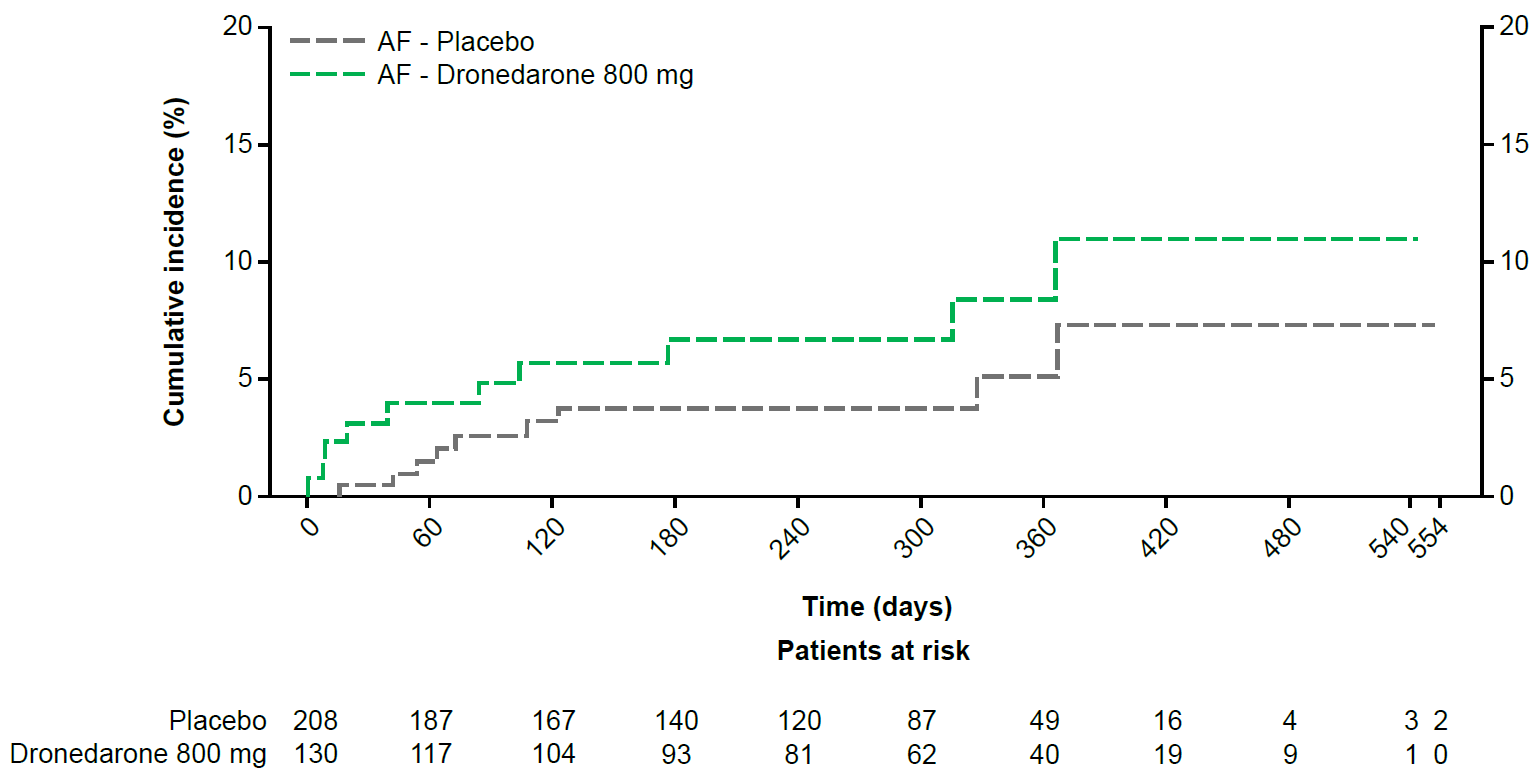


**Figure S2** Landmark analysis in patients in (A) SR and (B) AF at 12 months showing cumulative incidence of primary composite outcome from month 12 for dronedarone vs. placebo in patients with early AF. Kaplan–Meier cumulative incidence curves are shown. ECG rhythm status determined at month 12. Early AF defined as first known AF onset within ≤12 months. AF, atrial fibrillation/atrial flutter; ECG, electrocardiogram; SR, sinus rhythm.

**
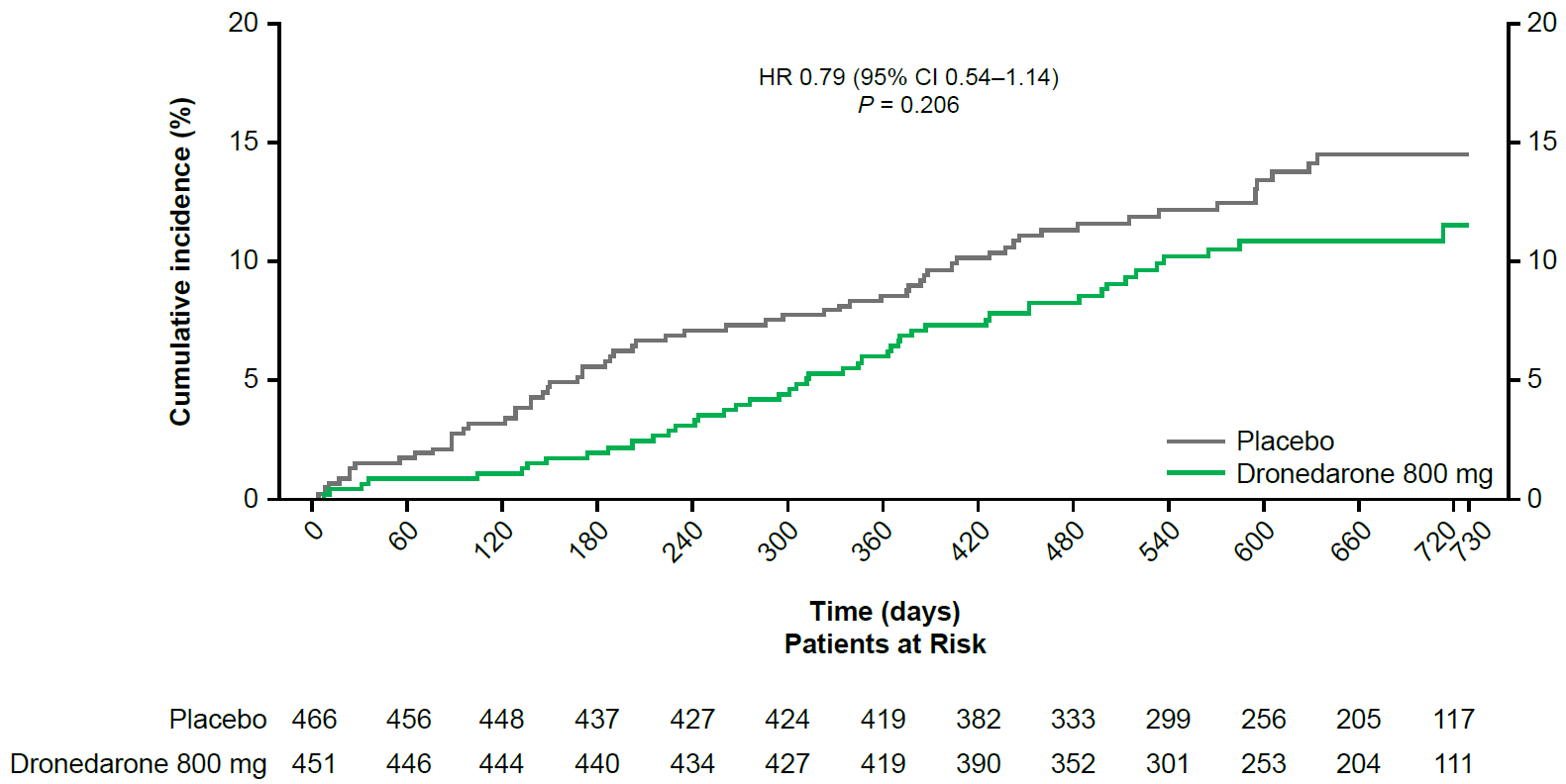
**

**Figure S3** Primary composite outcome (death from cardiovascular causes, stroke, or hospitalization due to worsening of HF or ACS) for dronedarone vs. placebo in patients with late AF. Aalen-Johansen cumulative incidence curves are shown. Late AF defined as first known AF onset >12 months.
ACS, acute coronary syndrome; AF, atrial fibrillation/atrial flutter; CI, confidence interval; HF, heart failure; HR, hazard ratio. Figure reproduced with permission from European Society of Cardiology.

**Supplementary References**

1. Hohnloser SH, Crijns HJ, van Eickels M, Gaudin C, Page RL, Torp-Pedersen C, et al. Effect of dronedarone on cardiovascular events in atrial fibrillation. N Engl J Med. 2009;360(7):668-78.

2. Kirchhof P, Camm AJ, Goette A, Brandes A, Eckardt L, Elvan A, et al. Early Rhythm-Control Therapy in Patients with Atrial Fibrillation. N Engl J Med. 2020;383(14):1305-16.

3. Kirchhof P, Breithardt G, Camm AJ, Crijns HJ, Kuck KH, Vandas P and Karl Wegscheider. Improving outcomes in patients with atrial fibrillation: rationale and design of the Early treatment of Atrial fibrillation for Stroke prevention Trial. *Am Heart J.* 2013;166(3):442-448.

4. Behnoush AH, Khalaji A, Naderi N, Ashraf H, von Haehling S. ACC/AHA/HFSA 2022 and ESC 2021 guidelines on heart failure comparison. ESC Heart Fail. 2023;10(3):1531-44.
